# Bacteria from Animals as a Pool of Antimicrobial Resistance Genes

**DOI:** 10.3390/antibiotics6020012

**Published:** 2017-06-06

**Authors:** Maria Angeles Argudín, Ariane Deplano, Alaeddine Meghraoui, Magali Dodémont, Amelie Heinrichs, Olivier Denis, Claire Nonhoff, Sandrine Roisin

**Affiliations:** 1 National Reference Centre—*Staphylococcus aureus*, Department of Microbiology, Hôpital Erasme, Université Libre de Bruxelles, Route de Lennik 808, 1070 Brussels, Belgium; Ariane.Deplano@erasme.ulb.ac.be (A.D.); Alaeddine.Meghraoui@erasme.ulb.ac.be(A.M.); Magali.Dodemont@erasme.ulb.ac.be (M.D.); Amelie.Heinrichs@erasme.ulb.ac.be (A.H.); odenis@ulb.ac.be (O.D.); Claire.Nonhoff@erasme.ulb.ac.be(C.N.); Sandrine.Roisin@erasme.ulb.ac.be (S.R.); 2 Ecole de Santé Publique, Université Libre de Bruxelles, Avenue Franklin Roosevelt 50, 1050 Bruxelles, Belgium

**Keywords:** *mec*, *cfr*, *mcr*

## Abstract

Antimicrobial agents are used in both veterinary and human medicine. The intensive use of antimicrobials in animals may promote the fixation of antimicrobial resistance genes in bacteria, which may be zoonotic or capable to transfer these genes to human-adapted pathogens or to human gut microbiota via direct contact, food or the environment. This review summarizes the current knowledge of the use of antimicrobial agents in animal health and explores the role of bacteria from animals as a pool of antimicrobial resistance genes for human bacteria. This review focused in relevant examples within the ESC(K)APE (*Enterococcus faecium*, *Staphylococcus aureus*, *Clostridium difficile* (*Klebsiella pneumoniae*), *Acinetobacter baumannii*, *Pseudomonas aeruginosa*, and *Enterobacteriaceae*) group of bacterial pathogens that are the leading cause of nosocomial infections throughout the world.

## 1. Introduction

The discovery of antimicrobial agents in the mid-twentieth century revolutionized the management and therapy of bacterial infections. Infections that would normally have been fatal became curable. Ever since then, the antimicrobial agents have saved the lives of millions of people. However, these gains are now seriously jeopardized by the rapid emergence and spread of antimicrobial-resistant bacteria [1]. Antimicrobial resistance (AMR) is a major health problem rapidly spreading across the world. The *Review on Antimicrobial Resistance* report [2] estimates that at least 700,000 annual deaths are due to infections by drug-resistant strains of common bacterial infections, human immunodeficiency virus (HIV), tuberculosis and malaria. Numbers suggested that up to 50,000 lives are lost each year due to antibiotic-resistant infections in Europe and the US alone [2]. The inappropriate use of antibiotics in food animals, as well as in the medical practice has potentiated the risk of untreatable infections. Due to the free movement of people and goods between countries, and the intensive international transport of livestock, the problem of AMR is becoming by nature a global problem. Moreover, the AMR emergence is accompanied with a decline in the discovery of new antimicrobial agents. It has been estimated that most of the antibiotics used presently for common human and animal infections will be useless within five to ten years, turning back the clock to the pre-antibiotic era [1].

Antimicrobial agents are principally used for therapy and prevention of human and animal diseases, but they are still used in some countries for growth-promotion in food animal productions [3]. Their indiscriminate use has contributed to the emergence of bacterial resistance, in hospitals, community and livestock settings. AMR may spread from animals to humans and vice versa; directly by the spread of the resistant bacteria or indirectly by the spread of resistance genes from animal bacteria to human bacteria. In this manuscript, we overview the current knowledge about the use of antimicrobial agents of critical importance in veterinary medicine, and investigate the potential of bacteria from animals as an AMR-gene reservoir. We have also underlined some resistance genes that were firstly described in bacteria from animals and later were found in human bacteria. This review focused in relevant examples within the ESKAPE (*Enterococcus faecium*, *Staphylococcus aureus*, *Klebsiella pneumoniae*, *Acinetobacter baumannii*, *Pseudomonas aeruginosa*, and *Enterobacter* spp.) or ESCAPE (*E. faecium*, *S. aureus*, *Clostridium difficile*, *A. baumannii*, *P. aeruginosa*, and Enterobacteriaceae) bacterial pathogens that are the leading cause of nosocomial infections throughout the world [4,5].

## 2. Use of Antimicrobials in Animal Health

Antimicrobial agents play a key role in the treatment of bacterial infections in human and veterinary medicine. In fact, AMR has been considered the quintessential One Health issue [6]. This One Health approach recognizes that the human health is connected to the animal health and the environment [7].

**Figure 1:**
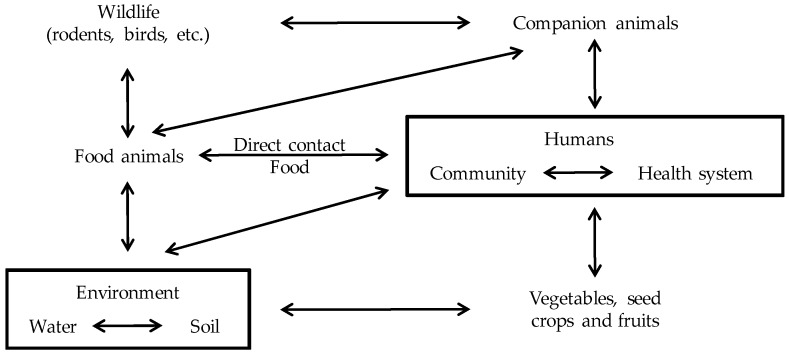
Interactions between groups. Antimicrobial-resistant bacteria can spread to humans either by the food supply, direct contact with food or companion animals or, more indirectly, through environmental pathways, including waterways, soils and vegetables contaminated with human or animals waste, and vectors such as rodents, insects, and birds. Based on da Costa et al. [8] and McEwen et al. [9] with modifications.

The use of antimicrobials in veterinary medicine creates a selective pressure for the emergence of antimicrobial resistant bacteria, including animal pathogens, human pathogens that have animal reservoirs and commensal bacteria from animals [8] The bacteria selected by this pressure can spread to humans either by direct contact with animals or food products, or indirectly via environmental pathways and/or non-food producing animals [8] (Figure 1).

The antimicrobial use in animals selects for AMR in commensal and zoonotic bacteria [9]. Soil treated with manure represents a “hot spot” of bacteria carrying AMR-genes [10]. However, soil itself is also a natural reservoir for antimicrobial-resistant bacteria [10]. The fecal wastes from animals contaminate groundwater, streams and other waterways, contributing to the spread of bacteria carrying AMR-genes [9]. Human wastes from homes, hospitals and offices also contribute to contaminate rivers and waterways with antimicrobial-resistant bacteria [9]. In fact, treated wastewater and lake water have been shown to contain AMR-genes and antimicrobial-resistant bacteria [10]. Soils and irrigation water are contamination sources for vegetables and fruits, in which resistant bacteria have been detected [10]. Antimicrobial-resistant bacteria may also spread between farms via infected carrier animals, companion animals or wildlife vectors [9]. Finally, there is a flow of patients and bacteria between community and hospital environments (Figure 1).

These complex transmission routes within farm animals, between farm animals and humans and the transfer of AMR-genes among bacteria, make it challenging to prove whether a reservoir of AMR-genes in livestock poses a risk for animal or human health [10]. However, some antimicrobial-resistant bacteria are zoonotic agents or can colonize and/or infect several hosts. In this sense, the current approach to evaluate the reservoir of AMR-genes in farm animals is to study the AMR-level of commensal bacteria and zoonotic agents in healthy farm animals and slaughter [10]. Reports from the European Food Safety Authority (EFSA) and the European Centre for Disease Prevention and Control (ECDC) monitoring AMR in animals are increasing. Yet, there are some limitations in the current data, and Thanner et al. [10] have recently suggested a voluntary monitoring program by researchers.

In order to underline the importance of the current available antimicrobials classes, the World Health Organization (WHO) started categorizing the most important antimicrobials in human medicine. The last revision of the list was done in 2016 [11]. The importance of each antimicrobial group is based on two criteria: C1, “the antimicrobial class is the sole, or one of limited available therapies, to treat serious bacterial infections in people”; and C2, “the antimicrobial class is used to treat infections in people caused by either: (i) bacteria that may be transmitted to humans from nonhuman sources, or (ii) bacteria that may acquire resistance genes from non-human sources”. Antimicrobials that meet both criteria are considered “critically important” in human medicine, antimicrobials that meet one of the criteria are considered “highly important”, and antimicrobials that meet none of the two criteria are considered “important” [11].

Similarly to the WHO, the World Organization for Animal Health (also named *Organisation mondiale de la santé animale*, OIE) [12] has developed a list of the antimicrobial agents of veterinary importance [13]. Since in veterinary medicine, many different species have to be treated the criteria to classify the antimicrobials were different than for the human medicine. The OIE criteria were based on a questionnaire prepared by the ad hoc group, which was sent to the OIE delegates of all member countries and international organizations which had signed a co-operation agreement with the OIE. The responses were analyzed by the ad hoc group and discussed in some international committees. The criterion C1 was based on the response rate to the questionnaire: “This criterion was met when a majority of the respondents (more than 50%) identified the importance of the antimicrobial class in their response to the questionnaire”. The criterion C2 was based on the treatment of each serious animal disease and the availability of alternative antimicrobial agents: “This criterion was met when compounds within the class were identified as essential against specific infections and there was a lack of sufficient therapeutic alternatives”. Similarly to the WHO list, antimicrobials that meet both criteria are considered “critically important” in veterinary medicine, antimicrobials that meet one of the criteria are considered “highly important”, and antimicrobials that meet none of the two criteria are considered “important”.

After the ban of antimicrobial growth promoters, antimicrobial agents are still allowed with veterinary prescription [10]. Around 37% of the antimicrobials (mainly ionophores) used in food animal production do not have equivalent drugs used for human therapeutic purposes [14]. Similarly, tetracyclines, that are not considered a first-line antimicrobial therapy in human medicine, make up another 44% of total antimicrobial used in animal agriculture [14]. While not all antimicrobial agents used in animal health are used in human medicine, most antimicrobials used in food animals are analogs to those used in human medicine [10]. In both WHO and OIE lists, substances belonging to certain groups (aminoglycosides, cephalosporins of third generation, macrolides, penicillins, and quinolones) were considered critical important antimicrobial groups [11,13]. In fact, some specific antibiotics are critically and/or highly important in both human and veterinary medicine (Table 1). Interestingly, the antibiotic streptomycin is also used in plant agriculture in the prevention of fire blight disease in apple and pear tree caused by the phytopathogenic *Erwinia amylovora* [10].

The use of antimicrobial agents in human medicine is restricted to therapy and prophylaxis. However, the antimicrobials in farm animals have therapeutic, prophylactic, metaphylactic and sub-therapeutic uses [9,15]. Therapeutic treatments are planned for individual animals that are diseased, but in food animal productions it is often more efficient to treat entire groups by medicating feed or water [9,15]. This metaphylactic use is particularly common and it implies the use of antimicrobials in the whole herd or flock for disease prophylaxis and/or therapy in case of presence of clinical illness in one individual of the group [3,15]. Moreover, for some animals (poultry and fish) this mass medication is the only feasible means of treatment [9]. This metaphylactic use results in the frequent exposition of entire groups of animals, healthy and diseased, to antimicrobial agents [10]. Prophylactic antimicrobial treatments are typically used during high-risk periods for infectious disease such as after weaning or transport [9]. Antimicrobials may also be administered in relatively low (sub-therapeutic) concentrations to food animals to promote growth and to enhance feed efficiency [9,15]. A ban on the use of growth promoters was implemented in Europe in 2006, but it has not led to any consistent decrease in antimicrobials consumption since this ban has been compensated by metaphylactic and prophylactic uses [3].

In addition to antimicrobials, metals compounds, such as zinc and copper, are also used to supplement animal (mainly in pigs) feed for the prevention of post-weaning diarrhea and the stimulation of growth. Resistance to these compounds is often associated to resistance to antimicrobial drugs such as methicillin in staphylococci, or macrolides and glycopeptides in enterococci [16]. It has been shown that these metal resistance genes are frequent in animal-associated bacteria [16,17,18,19]. The use of antimicrobials, biocides and metal compounds in animal productions in sub-therapeutic doses and with long exposure periods, may promote that bacteria fix genes that confer AMR [6,10]. These resistance genes can subsequently be transmitted to human-adapted pathogens or to human gut microbiota via people, contaminated food or the environment [6,10].

The multiple pathways involved in AMR-genes dissemination and exchange within the agriculture, the environment and the food processing industry (Figure 1) make difficult to track the movement of these genes in vivo [10]. In this regard, Thanner et al. [10] underlined some gaps regarding our current knowledge about AMR in plant and animal agriculture, and proposed a worldwide surveillance program of soil, plants, animals, water and wastewater treatment plants using the same methods that for AMR monitoring of human hospitals isolates. As for AMR in human bacteria, a better knowledge of the AMR in animal bacteria will help to achieve an effective and controlled use of antimicrobials in animals, thereby avoiding the dissemination of known and novel AMR-genes [10].

## 3. Presence of AMR-Genes in Animals: The Metagenomics Evidence

Although some bacterial species (such as *Mycobacterium tuberculosis* and *Streptococcus pneumoniae*) are specialist human pathogens, a larger number of species are opportunistic pathogens (such as *Escherichia coli*) causing disease in humans and other hosts including livestock and wildlife species, and are also present in the wider environment [3]. The interplay of these ecologies is important, since animals and the environment represent a major AMR reservoir. Through evolution, microorganisms have synthetized antibiotics and/or develop resistance methods for microbial competition in the environment [20]. In fact, AMR-genes have been found in soils not exposed to antimicrobials [21,22]. However, the human activity is increasing and changing this environmental resistome [20]. Indeed, animal microbiomes have acquired genes over years of exposure to antimicrobial agents and heavy metals compounds used as therapeutics, metaphylactics, prophylactics and growth promoters [3,23].

Current studies based on metagenomics and/or real-time polymerase chain reaction (PCR) approaches have given diverse results regarding the human, animal and environmental resistome. These novel technologies offer the possibility to elucidate the presence of AMR-genes in human, animal and environmental microbiomes and to identify the factors causing their persistence, selection and spread [10].

Some studies have suggested that human and animal microbiomes are different [21]. Agga et al. [21] compared environments related to animal (cattle and swine) and municipal (human) waste and saw that antimicrobial-resistant bacteria populations associated with animal agriculture were distinct from those associated to human activity. However, regarding the gene content, 25 out of 61 unique AMR-genes identified were common between municipal waste and animal samples. The half of the AMR-genes detected in another study [24], were only found in external environments. These genes from external environment microbiomes were mainly related to biocide and metal resistance [24]. Human microbiota had the highest abundance and diversity of AMR-genes, and the lowest taxonomic diversity [24]. Nevertheless, it was seen that tetracycline resistance genes dominated in both human and animal microbiomes [24]. Moreover, 20.5% of the AMR-genes detected were found in human, animal and environmental samples [24]. A recent study based on the comparison of published data on metagenomics made similar conclusions [25]. This study showed that the environment is a reservoir of the basic forms of resistance genes (such as *bla*_TEM_), while both the human and mammalian gut microbiomes contained the widest diversity of clinically relevant resistance genes [25].

Some studies have identified AMR-genes in animal samples regardless of the antibiotic exposure. Sequence-based metagenomics analysis of conventionally raised cattle without therapeutic antibiotics exposure revealed that 3.7% of the sequences encoded resistance to antibiotics and toxic compounds, and nearly half of these genes encoded multidrug resistance efflux pumps [26]. A similar metagenomics study in chicken ceca, revealed that around 2% of the sequences encoded resistance to antibiotics and toxic compounds [27]. At least one quarter of these genes were related to tetracycline and fluoroquinolones resistance [27]. Studies in swine fecal samples revealed the existence of at least 149 AMR-genes in non-medicated animals [28,29].

Although non-exposed animals may already carry bacteria with resistance genes, some studies have underlined that their resistome can change after antibiotic exposure. Diverse studies have shown that antibiotic treatment increased diversity of antibiotic resistance genes [28,29]. Moreover, some enriched genes, such as an aminoglycoside O-phosphotransferase, confer resistance to antibiotics that were not administered, demonstrating the potential for indirect selection of resistance to classes of antibiotics not fed [28]. The effects of administering sub-therapeutic concentrations of antimicrobials to beef cattle were investigated in a recent study, showing that the antimicrobial treatment differentially affected the abundance of certain resistance genes in fecal deposits, but not their persistence [30]. In other study, the administration of the third generation cephalosporin ceftiofur in dairy cows increased the β-lactam and multidrug resistance genes in feces [31]. Nevertheless, another study identified approximately the same number (21–26) of unique AMR-genes in manure samples of four dairy cows despite different prior exposure to antibiotics [32].

Some metagenomics studies have underlined the dominance of tetracycline resistance genes in animal microbiomes [24,33]. These results may be partially explained by the historical and current exposure to tetracyclines in the animal husbandry. Yet, a metagenomics study in pigs reared in an antibiotic-free environment revealed also the presence of diverse tetracycline resistance genes including novel genes, as well as a gene [*tet*(40)] not previously observed outside the human gut microbiome [34].

Although the metagenomics studies seem promising, they have some limitations. Since, some annotated genes (ex. efflux pumps) that confer AMR, perform other basic functions, more research on gene expression and functional analysis is needed to determine whether these genes can confer phenotypical resistance to antimicrobials [23]. Moreover, the sequence-based metagenomics approach does not provide information about the genetic context [23]. Nevertheless, some promising plasmid metagenomics studies have showed a broad dissemination of plasmid carrying AMR-genes in pig and bovine samples [35,36]. The functional genomics study about the tetracycline resistome of the pig gut has shown that most of the genes resided on putative mobile genetic elements (MGEs), which may contribute to the maintenance and dissemination of antibiotic resistance in antibiotic-free environments [34]. Nevertheless, other studies have probed that the presence of MGEs is also affected by antibiotic exposure [31,37]. It has been seen that the antimicrobial exposure increased the abundance of phage integrase-encoding genes (that may carry virulence or AMR-genes) in the viromes of swine, demonstrating the induction of prophages with antibiotic treatment [37]. In the study based on the administration of ceftiofur in dairy cows, an increase in gene sequences associated with phages, prophages, transposable elements and plasmids was observed [31].

Some metagenomics studies have proven that the animal microbiome is different of the human microbiome, but, interestingly, their shared a part of their resistome. Acquired resistance genes seem disseminated in the absence of selective pressure, but their abundance is affected by antibiotic exposition. These findings confirm that continue antimicrobial selective pressure in both humans and animals may benefit the dissemination of acquired resistance genes [23]. As other authors have concluded, a prudent use of antibiotics in human and veterinary health is needed to slow down the AMR spread and prevent the emergence of novel AMR-genes [23].

## 4. AMR-Genes in Gram-Positive Bacteria from Animals

Three Gram-positive species are considered members of the ESC(K)APE group: *C. difficile*, *E. faecium*, and *S. aureus.* This section focuses on the current knowledge about these pathogens regarding their AMR in animals, particularly on the genes common within these species (Table 2).

### 4.1. Clostridium difficile

*C. difficile* is an ubiquitous environmental organism widespread in rivers, lakes and soils. It is also found in the hospital environment where it is difficult to eradicate, as well as in meat products and animals including diverse species (calves, ostriches, chickens, elephants, dogs, horses and pigs) [49]. Although there are some current genomic updates of the clostridial phylogeny [50,51], in this review we use the classical nomenclature of *Clostridium difficile*.

*C. difficile* is recognized as the major cause of healthcare antibiotic-associated diarrhea [46]. Over the last decade, an alarming increase in incidence of *C. difficile* infection was observed across the USA, Canada and Europe, and it has been associated with the emergence of the highly virulent (hypervirulent) clone BI/NAP1/027 [named according to its restriction enzyme analysis (REA), pulsed-field gel electrophoresis (PFGE) and PCR Ribotype (RT)] [46,52]. This dramatic increase in *C. difficile* infections was associated to the fluoroquinolone resistance of this clone [52]. Fluoroquinolones are broad-spectrum antimicrobials highly effective for the treatment of bacterial infections in animals and humans. Most RT027 isolates harbored mutations in the quinolone-resistance determining region (QRDR) of the DNA gyrase subunit gene, *gyrA*, that confer resistance to fluoroquinolones [52]. Nevertheless, clinical *C. difficile* strains acquire fluoroquinolone resistance due to alterations in the QRDR of either GyrA or GyrB DNA gyrase subunits [46].

*C. difficile* infection has also emerged as a cause of diarrhea in the community, especially in populations previously considered at low risk, such as young people, antibiotic-naive patients or people without healthcare exposure [46]. Studies in the community indicated that up to 13% healthy human subjects are asymptomatically colonized with *C. difficile* [52]. In addition to RT027, a number of emergent hypervirulent RTs have recently been identified, notably the hypervirulent RT078 recognized as infection cause in hospitals, the community and animals [46]. The use of fluoroquinolones in the pork industry may have also contributed to the emergence of the multidrug-resistant RT078 [53]. In a study by Keessen et al. [53], most human and porcine isolates were resistant to ciprofloxacin (96%), and some were also resistant to moxifloxacin (16% for both human and porcine isolates). Resistance to moxifloxacin in this study was associated with a *gyrA* mutation [53]. Moreover, the use of fluoroquinolones [ciprofloxacin in humans, and enrofloxacin in pigs] was significantly associated with isolation of moxifloxacin-resistant isolates in both populations [53]. The authors proposed that the increased fluoroquinolone use could have contributed to the spread of *C. difficile* RT078 [53].

RT078 is commonly isolated from swine and other food animals [52]. It is likely that RT078 is an important pathogen of piglet diarrhea worldwide [49,54]. Molecular genotyping has suggested that RT078 isolates of human and swine origin are highly related and may therefore represent a potential zoonotic transmission [52]. Several studies have suggested that transmission from swine to humans may occur in farms or in large integrated swine operations, and moreover, the aerial dissemination of *C. difficile* from pig farms has been shown [52,54]. Due to the increased incidence of *C. difficile* infection outside the hospital environment and the presence of the same genetic lineage in food animals and its products, some authors have suggested that *C. difficile* may be considered as a foodborne pathogen [52]. Although RT078 is predominant in studies on swine, other RTs have been described in animals [52]. For example, RT046 has been found in both piglets and humans in Sweden [55].

AMR in *C. difficile* has been less intensively investigated than in other Gram-positive pathogens (such as *S. aureus*). In fact, few AMR-genes [such as *erm*(A), *erm*(B), *tet*(M), *tet*(44), *tet*(W), *ant(6)-Ib*, *catD* and *cfr*(B)] have been characterized in *C. difficile* isolates [46]. Resistance to cephalosporins is still uncharacterized, although most clinical *C. difficile* strains are resistant to these antibiotics. Similarly, some *C. difficile* human and animal isolates with reduced susceptibility to metronidazole have been found, although the resistance mechanism is not completely understood [46].

The most widespread mechanism of resistance to the antibiotics of the macrolide-lincosamide-streptogramin B (MLS_B_) group in *C. difficile* is ribosomal methylation due to the erythromycin ribosomal methylases (*erm*) genes of class B [46]. It is to note, that *erm*(B) is the *erm* gene with the widest bacteria host range, and it has been found in both Gram-positive and Gram-negative bacteria, aerobic, and anaerobic genera and in most ecosystems [56]. However, *erm*-negative *C. difficile* strains resistant to both erythromycin and clindamycin or only to erythromycin have been also described [46]. Alterations in the 23S rRNA or ribosomal proteins (L4 or L22) have been found in some of these strains, but the presence of these changes in susceptible isolates had excluded their role in resistance [46]. Interestingly, a multidrug resistant *cfr*-like gene, *cfr*(B), that modifies the 23S rRNA has been recently found in clinical *C. difficile* isolates [57,58] (see Section 4.3.2).

The *tet*(M) gene is the most frequent tetracycline resistance determinant in *C. difficile* isolates [46]. Interestingly, most RT078 isolates carry the transposon *Tn*916 harboring *tet*(M) [52]. Nevertheless, other *tet* genes [*tet*(W), *tet*(44)] have been identified in *C. difficile* [46,59]. The *tet*(W) gene has been found together with *tet*(M) in *C. difficile* isolates recovered from animals and humans [46]. The *tet*(44) gene has been associated to the transposon *Tn*6164 in human and environmental isolates [46,59].

### 4.2. Enterococcus faecium

Enterococci are commensal bacteria of the gastrointestinal tract of mammals and other animals that can also be detected in the environment [60,61]. In adult people, enterococci account for about 1% of the intestinal microbiota, being *E. faecium* and *Enterococcus faecalis* the most prevalent enterococci in the human gastrointestinal tract [61]. These species are opportunistic human pathogens that are implicated in life-threatening hospital acquired infections such as bacteremia and infective endocarditis [60,61].

Enterococci are intrinsically resistant to a number of first-line antimicrobial agents. They have resistance against cephalosporins and cotrimoxazole, as well as low-level resistance to β-lactams and aminoglycosides [60]. Moreover, clinical and animal *Enterococcus* isolates with resistance to other antimicrobials such as macrolides, tetracyclines, streptogramins and glycopeptides have been described [60,62]. Antimicrobials such as linezolid, daptomycin and tigecycline can be used in the treatment of enterococcal infections [63,64]. Nevertheless, resistance against these former antimicrobials has been reported, mainly in the clinical setting [63,64].

Nowadays, the vancomycin-resistant enterococci (VRE) pose a major therapeutic challenge due to their difficult treatment [61]. VRE were found in the hospital setting in the 80s [61,64], while the first description of animal reservoirs of vancomycin-resistant *E. faecium* was published in 1993 [65]*.* Later on, VRE have been described in diverse animals and environmental sources [60]. As vancomycin has not been used in veterinary medicine, it was hypothesized that the use of another glycopeptide, the avoparcin, as additive in farm animals feed, has influenced the emergence of VRE in animals since the 70s [60]. This correlation has been criticized and several studies have shown that vancomycin-resistant *E. faecium* persisted in animals for an extended time after the banning of avoparcin in the 90s [54,60]. However, this persistence has been related to co-selection with other antimicrobials (such as tetracycline or the macrolide tylosine) and metals (such as copper sulphate) [54,60]. It has been suggested that VRE strains are predominately host-specific, being hospital isolates genetically different from animal strains [60]. However, some research, based on multilocus sequence typing (MLST) showed that certain isolates from diverse clonal complexes (CCs) are present in animals, healthy humans and patients [54,60,61]. Moreover, the same variants of *Tn*1546 carrying the glycopeptide-resistant gene *vanA* have been detected in enterococci from human and animal origin, underlying that *E. faecium* from animals can act as a donor of AMR-genes for other pathogenic enterococci [54,60].

Similar to glycopeptides, the use of other antimicrobials as grown promotors may have influenced the emerging of enterococci resistant strains in food animals. In 1999, the streptogramin combination quinupristin/dalfopristin (RP59500, Synercid) was approved for clinical use. The mixture of type B streptogramin (quinupristin) and type A streptogramin (dalfopristin) in a 30:70 ratio showed good activity against multiresistant *E. faecium* strains [66]. Nevertheless, before its approval for clinical use, in 1997, quinupristin/dalfopristin-resistant *E. faecium* isolates were found in both human patients and chicken samples [66]. The use of virginiamycin, a streptogramin licensed for growth promotion in animals (including chickens and other poultry), was associated to the development of resistance to streptogramin combinations by enterococci [66]. In this sense, diverse streptogramin A resistance genes [such as *vat*(D), *vat*(E), *vga*(D) and *vat*(H)] have been detected in *E. faecium* from animals in Europe, USA and/or Asia [60].

Regarding other antimicrobials, human and animal *E. faecium* isolates frequently harbored genes conferring resistance against aminoglycosides [*aph(3′)IIIa*], tetracycline [*tet*(M)], and macrolides [*erm*(B)] [61]. Recently, a novel gene, *optrA*, which encodes for an ABC transporter that confers resistance to oxazolidinones and phenicols has been found in *E. faecalis* and *E. faecium* of human and animal origin [67]. This gene has been found chromosomally and plasmid located in Enterococci [40], but also in *Staphylococcus sciuri* [41,68]. The *optrA*-carrying plasmids in enterococci have also other AMR-genes against antibiotics such as MLS_B_ [*erm*(A)-like)] and/or phenicol (*fexA*) [40]. Retrospective analysis of genome sequences has revealed that *optrA* has a wide dissemination in Gram-positive bacteria and it has also been found in diverse *Streptococcus* species (including *S. suis*, *S. agalactiae* and *S. pyogenes*) [69].

Some genes (*cfr* and *dfrK*) first discovered in animal-related staphylococci have recently been found in enterococci (see Section 4.3.2 and Section 4.3.3). The trimethoprim resistance gene *dfrK*, encoding a trimethoprim resistant dihydrofolate reductase, was recently detected in *E. faecium* [43] (see Section 4.3.3). The multi-resistance gene *cfr* encodes a RNA methyltransferase that modifies the 23S rRNA gene conferring resistance to ribosome-targeting antimicrobials [44]. It has been detected in *E. faecalis* from animals and humans, *E. thailandicus* from pigs and farm environment, *E. casseliflavus* and *E. gallinarum* from pigs [42,45]. A non-functional *cfr*, due to a deletion in the regulatory region upstream, has been detected together with *optrA* in a clinical *E. faecium* isolate [38]. As for *C. difficile*, a functional *cfr*(B) has recently been found in *E. faecium* clinical isolates [39] (see Section 4.3.2).

### 4.3. Staphylococcus aureus and Related Species

*S. aureus* is part of the normal and transit human microbiota, and it is usually present in the nasopharyngeal mucosa, but also in the skin and other corporal areas [70,71,72]. The general carrier rate in humans is estimated between 20 and 30 percent for persistent colonization [73]. *S. aureus* is an opportunistic pathogen, which produces a wide spectrum of diseases, ranging from minor’s skin to deep infections, as well as potentially-fatal diseases such as diverse invasive infections and toxin-mediated diseases [74,75]. As in humans, *S. aureus* is a commensal bacterium for animals but it is also able to cause diverse infections including abscesses, chondronecrosis, dermatitis, mastitis, pyaemia, osteomyelitis, pneumonia, septicemia and skin and wound infections [76].

Staphylococci of animal origin harbor a wide variety of AMR-genes [48,77,78,79,80,81,82,83]. Recent reviews by Wendlandt et al. [48,81] have underlined that at least 44 AMR-genes in staphylococci have been detected in both human and animal isolates (Table 3). Interestingly, some shared genes were firstly described in isolates from animal origin [such as *tet*(L), *erm*(T), *dfrK*, *fexA*, *cfr*] or possibly originated from *Staphylococcus* species related to animals (as the methicillin resistance gene *mecA*). Next sub-sections are focused on those genes originated from animal related staphylococci.

#### 4.3.1. *mec* Genes in Staphylococci: Origin and Reservoirs

One of the most important acquired resistance in staphylococci is methicillin resistance. This resistance is mainly due to the acquisition of the *mecA* gene, encoding a β-lactam low affinity penicillin binding protein (PBP) called PBP2a. This gene is carried on a MGE termed staphylococcal cassette chromosome *mec* (SCC*mec*). This MGE has been more extensively studied for *S. aureus*, since this is the most important *Staphylococcus* species for the human health. To date, eleven major SCC*mec* types carrying *mecA* have been described in methicillin resistant *S. aureus* (MRSA), which have been assigned on the characterization of its two essential components the *mec* and *ccr* complexes [84,85,86]. The *mec*-gene complex corresponded to the *mecA* operon variants, which can include functional and/or truncated regulatory genes. The *ccr* complex includes the recombinase(s) involve in the excision and integration of the element into the chromosome. The remaining regions of the SCC*mec* on which subtypes are based are called junkyard or joining regions, and they can contain MGEs, as well as heavy metal [arsenate (*ars* operon), cadmium (*cadD-cadX*), cadmium and zinc (*ccrC*), copper (*copB*), mercury (*mer* operon)] and other AMR (amynoglicosides (*aadD*, *aad9/spc*, *aacA-aphD*), bleomycin (*ble*), erythromycin [*erm*(A)], tetracycline [*tet*(*K*)]) resistance genes. Many non-typable SCC*mec* cassettes exist, and non-*S. aureus* staphylococci carry similar SCC*mec* and novel *mec-ccr* combinations as those found in *S. aureus* [17,87,88,89,90]. This suggests horizontal transfer and recombination events within Staphylococci species. In fact, the *mecA* is widely distributed among methicillin resistant coagulase negative Staphylococci (MRCoNS) [17,87,88,89,90,91].

Closed related *mecA* allotypes have been described in members of the *S. sciuri* group [98,99,100]. The members of the *S. sciuri* group are mainly considered commensal animal-associated bacteria with a broad range of hosts, although they have also been found in the environment and occasionally causing disease in humans and other hosts [98,99,100]. The *mecA* variants found in members of the *S. sciuri* species group are chromosomal located without being part of a SCC*mec*, and are usually phenotypically susceptible to β-lactams or show a heterogeneous resistance [91]. In this sense, a member of the *S. sciuri* group, the commensal animal-related *S. fleurettii*, has been suggested as the highly probable origin of the *mecA* gene [101]. The *mecA*-containing regions of *S. fleurettii* strains recovered from animals or its products are similar to the *mec* region of the *S. aureus* SCC*mec* type II. Moreover, the analysis of the corresponding gene loci region (with *mecA* gene homologues) of *S. sciuri* and *S. vitulinus* (which evolved from a common ancestor with that of *S. fleurettii*), probed that the *mecA* gene of *S. fleurettii* descended from its ancestor and was not recently acquired [101]. These findings suggested that the SCC*mec* in *S. aureus* was generated by acquiring this intrinsic *mecA* region of *S. fleurettii*.

Recently, two *mecA* gene homologs (*mecB* and *mecC*) have been discovered [102,103,104]. The gene *mecB* (formerly named *mecAm*) was found in 2009 in a *Macrococcus caseolyticus* isolated from a chicken [102]. This gene is located in a typical *mec* operon (*blaZb-mecB-mecR1b-mecIb*) with similar regulatory genes but also accompanied of a *blaZ* homologue encoding for a putative β-lactamase [85]. The *mecB* operon has been found on a transposon (*Tn*6045) either plasmid or chromosomal located [102], as well as in SCC*mec* elements [102,105]. Nowadays, *mecB* has not been detected in staphylococci. However, there is a potential risk of transmission since staphylococci and macrococci are closely related members of the same family (*Staphylococcaceae*). Moreover, these bacteria share a common niche, since *M. caseolyticus* is a commensal bacterium colonizing animal skin as staphylococci. Additionally, this *mec* variant is located in different MGEs, including a plasmid, which is a more mobile vehicle within bacteria than the SCC*mec* [85].

The gene *mecC* (formerly named *mecA*_LGA251_) was discovered in 2011 in *S. aureus* by two different working groups [103,104]. It was described from isolates originating from mastitis in cows and from humans in the United Kingdom, Ireland and Denmark [103,104]. Even if this gene was first described in 2011, a retrospective analysis in Denmark has shown that *mecC*-positive MRSA strains have been circulating before [106]. The *mecC* gene has a wide distribution and positive strains have been found in humans from both infection cases and carriage state, as well as in animals including livestock (dairy cattle, beef cattle, sheep, farmed rabbits), companion (cats, dogs, guinea pigs), wildlife (birds, mammals) and zoo (mara) animals [91,107]. Generally, there is a low occurrence of *mecC*-positive human isolates (ex. less than 1% in clinical MRSA from Belgium, [108]). This gene has mainly been associated with *S. aureus* lineages related to infections and colonization in animals [91]. The gene *mecC* in *S. aureus* is located on the SCC*mec* XI, which as few other SCC*mec* types also carried heavy metal resistance genes [104]. *mecC* homologues have been found in other staphylococci (*S. xylosus*, *S. sciuri*, and *S*. *stepanovicii*), associated to cassettes similar to SCC*mec* XI or to composite SCC*mec* together with *mecA* [109,110,111]. New *mecC* allotypes have been identified: *mecC1* in *S. xylosus* related to bovine mastitis [109] and *mecC2* in *Staphylococcus saprophyticus* from common shrew [112].

#### 4.3.2. The Multi-Resistance Gene *cfr*

Linezolid, the first member of the oxazolidinone class of antibiotics, is considered to be a last-resort antimicrobial for the treatment of infections caused by VRE, MRSA and penicillin-resistant pneumococci [45]. Several mechanisms conferring linezolid resistance have been described in staphylococci, including point mutations in genes encoding 23S rRNA and mutations in ribosomal proteins L3, L4 and L22 [113,114]. In 2000, the gene *cfr* was identified in a bovine *S. sciuri* recovered in 1997 [115]. This gene encodes an RNA methyltransferase that modifies the 23S rRNA gene conferring combined resistance to phenicols, lincosamides, oxazolidinones, pleuromutilins and streptogramin A antimicrobials (known as PhLOPSA_A_ phenotype). It also confers decreased susceptibility to the 16-membered macrolides (josamycin and spiramycin) [45]. Afterwards, this gene has been identified in methicillin-susceptible *S. aureus* (MSSA), MRSA, various coagulase negative staphylococci (CoNS) and in the coagulase-variable *S. hyicus* [45].

Although linezolid resistance mediated by *cfr* is not frequent in the clinical environment [116], reports of clinical outbreaks by *cfr*-containing *S. aureus* strains have been reported [117,118]. Recently, a clinical case due to a *cfr*-positive livestock-associated (LA-) MRSA CC398 has been described [119]. This gene has been found in important MRSA pandemic lineages such as the sequence type (ST)22/SCC*mec* IV, the Panton-Valentine leukocidin (PVL) positive ST8/SCC*mec* IV/USA300 or the ST125-MRSA-IVc [114,120,121]. It has also been detected in clinical strains of *S. capitis* [122] and methicillin-resistant *S. epidermidis* (MRSE) [123].

In staphylococci from animals, the *cfr* gene has been identified in isolates from different sources including pigs (in *S. aureus*, *S. arlettae*, *S. cohnii*, *S. equorum*, *S. haemolyticus*, *S. hyicus*, *S. saprophyticus*, *S. sciuri*, *S. simulans* and *S. warneri*), bovine (in *S. aureus*, *S. lentus*, *S. sciuri* and *S. simulans*), poultry (in *S. arlettae*, *S. cohnii*, *S. equorum*, *S. rostri*, *S. sciuri* and *S. simulans*), and companion animals (in *S. pseudintermedius*) [45,81,124,125]. The *cfr* gene has been described as chromosomally and plasmid located, linked to specific insertions sequences (IS) [45]. Moreover, the genetic elements of staphylococci with *cfr* usually carried additional resistance genes, including β-lactam (*blaZ*), aminoglycosides [*aacA-aphD*, *aadD*, *ant(4′)-Ia*], aminocylitols (*spc*), bleomycin (*ble*), fosfomycin (*fosB*), MLS_B_ [*erm*(A), *erm*(B), *erm*(C), *erm*(33)], lincosamides-pleuromutilins-streptogramin A [*lsa*(B)], phenicol (*fexA*), tetracycline [*tet*(L)], oxazolidinones-phenicols (*optrA*) and/or trimethoprim (*dfrK*) resistance genes (Table 4). The immediate genetic environment within the diverse *cfr*-carrying elements is similar, therefore it has been suggested that a limited number of acquisition events explain the diversity seen among the plasmidic or chromosomal structures [45].

Recent studies have described the *cfr* gene or a variant in other Gram-positive (including *Bacillus* spp., *C. difficile*, *Enterococcus* spp., *Macrococcus caseolyticus*, *Jeotgalicoccus pinnipedialis* and *S. suis*) and Gram-negative (*Proteus vulgaris* and *E. coli*) species (Table 4). With the exception of *Clostridium* and *Enterococcus* isolates, the *cfr*-positive isolates of the other non-*Staphylococcus* genera were obtained exclusively from livestock and related farm environments [39,45,57,58].

In *Bacillus* spp., the gene *cfr* has been identified in three related plasmids from pig isolates from China, one (PBS-01) of these plasmids being previously reported in *S. cohnii* [45]. The *cfr*-carrying plasmid PSS-03 has been detected in both *M.* *caseolyticus* and *S. cohnii* [45], while the plasmid pJP1 or variants type pJP1-like have been found in *M.* *caseolyticus*, *J. pinnipedialis*, and *S. lentus* isolates [45]. Interestingly, some of these *cfr*-plasmids found in Gram-positive bacteria carried additional resistance genes against aminoglycosides (*aadD*, *aadY*), bleomycin (*ble*), MLS_B_ [*erm*(B), *erm*(C)] and/or phenicol (*fexB*). In fact, a new multiresistance plasmid pWo28-3 related to pJP1, harbouring *aacA-aphD*, *aadD*, *ble*, *optrA*, *cfr* and *fexA*, has been identified in *S. sciuri* [68]. The *cfr* has also been found associated to a novel IS (IS*Enfa5*) truncating a novel lincosamide resistance gene *lnu*(E) in the plasmid pStrcfr of *S. suis* [126].

Recently, the *cfr* variant gene, designated *cfr*(B), was identified in *C. difficile* and *E. faecium* clinical isolates [39,57,58]. The nomenclature and functionally of this gene has been extensively discussed [127]. The Cfr proteins detected in other Gram-positive (*Bacillus*, *Macrococcus*, *Jeotgalicoccus*, *Staphylococcus* and *Streptococcus*) and in Gram negative (*Proteus* and *Escherichia*) bacteria were indistinguishable or similar (99% identity) from the original *S. sciuri* Cfr protein [127]. However, the *cfr*(B) shared only 75% amino acid identity with the original Cfr protein [39,58,127]. Schwarz et al. [127] suggested that the protein encoded by *cfr*(B) was structurally distantly related to the original Cfr. However, further research proved that the *cfr*(B) product does function as a Cfr protein [58], and therefore may be considered as a variant.

Regarding *Enterococcus*, *cfr*-carrying plasmids have been described in *E. faecalis* from animals and humans, as well as in pig-related isolates of *E. thailandicus*, *E. casseliflavus* and *E. gallinarum* [42,45]. The *cfr* gene has also been detected chromosomally located in *E. casseliflavus* from pigs [42]. As mentioned in Section 4.2, a non-functional *cfr* gene has been detected together with *optrA* in clinical *E. faecium* isolates [38]. However, recently a *cfr*(B) gene sharing 99.9% sequence identity with the corresponding gene in *C. difficile* has been identified in *E. faecium* clinical isolates [39,128]. Deshpande et al. [39] described two copies of *cfr*(B) chromosomally located and embedded in a *Tn*6218 similar to the *cfr*-carrying transposon described in *C. difficile*. While the *cfr*(B) described by Bender et al. [128] was found in *Tn*6218-like elements possible linked to plasmids.

It has been shown that the gene *cfr* is functionally active in Gram-negative hosts [45]. Furthermore, it has been identified chromosomally located (inserted into the chromosomal *fimD* gene) in *P. vulgaris* from pigs, and in diverse plasmids in *E. coli* from pigs [45,130,131,132,133,134] or food of animal origin [135]. The IS26 appears to play an important role in the transfer of this multiresistance gene in Gram-negative bacteria, since it appears in the *cfr*-carrying plasmids detected in *E. coli* [45,130,131,132,133,134,135]. One of these *cfr*-carrying plasmids from *E. coli* of swine origin carried also the extended-spectrum-β-lactamase (ESBL) gene *bla*_CTX-M-14b_ [133].

#### 4.3.3. Other Genes in Animal-Associated *S. aureus*

*S. aureus* from animals can be related to various CCs [90,136,137]. However, animals are considered the main reservoir of the specific *S. aureus* lineage CC398, which has been the subject of numerous studies during recent years [18,19,82,138,139,140,141,142,143]. Studies based on the phylogenetic analysis of genome-wide single nucleotide polymorphisms (SNPs) supported the existence of two subpopulations in CC398: an ancestral human-adapted clade and an animal-associated clade [144,145]. Nevertheless, LA-MRSA CC398 isolates of the animal-associated clade are able to infect humans [119,145]. Analysis of genes present in CC398 has revealed numerous genes in common with clinical *S. aureus* and/or other staphylococci, but also some novel or rare resistance genes have been found in animal-related isolates from this lineage [79,81]. Some of these rare or novel genes are often on plasmids encoding for other AMR-genes such as the multi-resistance gene *cfr* (Table 5).

One interesting gene, found in animal-associated *S. aureus*, is the phenicol exporter gene *fexA*. It was first described in a bovine *S. lentus* isolate, but later it has been found in diverse plasmids of LA-MRSA CC398 from pigs, cattle and horses, as well as on plasmids from the also animal-related *S. aureus* CC9 [79,81]. This gene has been related to the non-conjugative transposon *Tn*558 that has been detected (partially or complete) in plasmids or in the chromosome of various CoNS of animal origin [79]. The *fexA* is co-located in plasmids with additional resistance genes (Table 5). Interestingly, the *fexA* gene has been described in *cfr*-carrying plasmids of clinical important MRSA pandemic lineages [114,120].

The tetracycline gene *tet*(L) was found initially in the 80s in diverse plasmids from *Bacillus*. In staphylococci, it was first described in the early 90s on a plasmid from a porcine *S. hyicus.* In addition, subsequently, it was detected on structurally diverse plasmids of staphylococci of animal origin [77,79]. This gene has been found co-located on plasmids from staphylococci with other additional antimicrobial and heavy metals resistance genes (Table 5). Regarding LA-MRSA CC398, the gene *tet*(M) is the most frequent tetracycline-resistant gene found in these isolates, although it is accompanied frequently by *tet*(K) and *tet*(L) [139]. The gene *tet*(L) has also been described in clinical *S. aureus* in the 90s [146], and recently in a *cfr*-carrying plasmid with additional antimicrobial genes [*dfrK* and *aadD*] in a clinical *S. aureus* ST125 strain [121].

The trimethoprim resistance gene *dfrK* was discovered co-located on a plasmid with *tet*(L) in LA-MRSA CC398 [79,81]. Similar to other genes in staphylococci, the *dfrK* has been found on diverse plasmids co-located with other antimicrobial and heavy metals resistance genes (Table 5). The *dfrK* gene is widely disseminated in LA-MRSA CC398 and it has been found in isolates from pigs, cattle and poultry [79]; but it has also been found chromosomally located on the transposon *Tn*559 of MSSA CC398 [147]. Additionally, the *Tn*559 carrying *dfrK* has been found in *E. faecium* [43].

The *erm*(T) gene has been previously identified in other Gram-positive bacteria (such as *Streptococcus* and *Lactobacillus*) [79]. In staphylococci, this gene has been originally found in animal-associated CC398, but further research suggested that it is particularly associated to the MRSA and MSSA CC398 from the ancestral human clade [79,149,150]. Nevertheless, a recent study comparing *erm*(T)-carrying plasmids from *S. aureus* ST398 from pig and humans, showed that these plasmids are quite similar and all carried additional tetracycline [*tet*(L)] and heavy metal (*cadD-cadX*, *copA*, *mco*) resistance genes [148]. The plasmid (pUR1902) recovered from a pig isolate carried also *aadD* as a plasmid (pUR2941) from a human isolate, while the remaining plasmid (pUR2940) from a human isolate additionally carried *erm*(C) and *dfrK* [148].

A recent review by Wendlandt et al. [81] underlined the presence of diverse multidrug resistance (MDR) genes co-located together or with other resistance genes on plasmids in staphylococci from animal origin. These genes included MLS_B_ [*erm*(A), *erm*(B), *erm*(C), *erm*(T), *erm*(33)], lincosamides-pleuromutilins-streptogramin A [*lsa*(E), *vga*(A), *vga*(C)] and PhLOPSA_A_ (*cfr*) resistance genes [81]. The main resistance mechanisms conferred by these MDR genes included target modification by methylation (*erm* and *cfr* genes) and active efflux via ABC transporters [*vga* and *lsa*(E) genes] [81]. Their location on plasmids, co-located with other AMR-genes, may potentiate their co-selection and persistence in animal staphylococci, and furthermore represent an important pool of resistance against critically and highly important antimicrobial agents [81].

## 5. AMR-Genes in Gram-Negative Bacteria from Animals

The Gram-negative members of the ESC(K)APE group are *A. baumannii*, *P. aeruginosa*, and the members of Enterobacteriaceae. A large number of antimicrobial resistance genes have been described in Gram-negative bacteria [44,47]. Since this area is extremely large and complex, in this section, we summarize part of the current knowledge about AMR in *A. baumannii*, *P. aeruginosa* and Enterobacteriaceae from animals, with special attention to the emergence of carbapenemase-producing Gram-negative bacteria in animals (Table 6) and the emergence of the colistin resistance.

### 5.1. Acinetobacter baumannii

*A. baumannii* is an important nosocomial pathogen that usually affects immunocompromised patients suffering from various underlying diseases [159]. Nosocomial infection with this bacterium has been associated with increased morbidity, mortality and health care costs [160]. It is responsible of hospital outbreaks, and it has a remarkable ability to survive for prolonged periods throughout hospital environments [160]. It is well established that the members of the genus *Acinetobacter* are ubiquitous microorganisms. However, *A. baumannii* as a highly prevalent microorganism in nature, is a misconception because the difficulties encountered in its identification [159,160]. In fact, *A. baumannii* is phenotypically and genotypically closely related to other *Acinetobacter* species (*A. pittii*, *A. nosocomialis* and *A. calcoaceticus*), making the species identification challenging [159].

*A. baumannii* has been related with community-acquired infections (single events or case series) [160], and has been isolated from various environmental locations: soils contaminated with petroleum hydrocarbons, vegetables, inanimate surfaces in contact with humans, manured agricultural soil, pig slurry and aquaculture environments [160]. Multi-susceptible *Acinetobacter* isolates were commonly found in the 70s–80s in soil and in the hospital setting, clinical *A. baumannii* being easily treated with common antibiotics during this period [20,159]. However, this bacterium has an extraordinary ability to upregulate or acquire resistance determinants, and nowadays infections with multidrug or even pandrug resistant isolates are increasing [159].

During the last decade, *A. baumannii* strains have also been isolated from animals, mainly causing outbreaks in veterinary clinics or hospitals [160]. *A. baumannii* strains have been isolated from diverse animals including ducks, pigeons, chickens, donkeys, rabbits, pets (cats and dogs), mules, livestock (cattle, caws, goats, pigs), horses, lice and arthropods [161]. In a study based on PFGE, it was seen that isolates from livestock were different than *A. baumannii* human strains [162]. Yet, isolates recovered from pig fecal samples harbored *bla*_OXA-51_, which has already been reported in human clinical isolates [162]. In the same study new *bla*_OXA-51_-like genes (*bla*_OXA-148_, *bla*_OXA-149_ and *bla*_OXA-150_), not previously detected in human isolates, were described in bacteria from cattle [162]. However, it is important to underline that the *bla*_OXA-23_ and *bla*_OXA-51_-like genes may be naturally occurring in *A. baumannii* [163]. On the other hand, the studies in companion animals have reported *A. baumannii* isolates genetically similar to the nosocomial European (also called International) clones I, II and III, suggesting its spread from humans to animals directly or via the environment [159,160,164,165].

Regarding the distribution of AMR-genes in *A. baumannii* in animals, some studies have reported the emergence of carbapenemase producing *A. baumannii* in livestock and companion animals [153,159] (Table 6). The *bla*_OXA-23_ is a wide distributed carbapenemase gene in *A. baumannii* isolates, which has been described recently in livestock and/or pets [151,153,154,159,165]. The *bla*_OXA-23_ gene has also been found in *A. lwoffi* from poultry [154]. In *A. baumannii* this gene has been found in STs not previously reported in humans, as well as in *A. baumannii* strains belonging to ST2, which has been previously related to hospitals outbreaks [151]. The *bla*_OXA-58_ has been found together with *bla*_OXA-23_ in *A. baumannii* from fowl [151]. Similarly, a *bla*_NDM-1_ positive *A. baumannii* isolate has been recovered from a pig suffering from pneumonia and sepsis [158]. This *bla*_NDM-1_ was harbored by a plasmid also carrying other AMR-genes [*aphA6*, *ble* and *msr*(E)-*mph*(E)] [158]. The *bla*_NDM-1_ has been found in other *Acinetobacter* species [*A. junii* (from a pig farm) and *A. calcoaceticus* (from around a cow farm)] recovered from environmental samples and farm animals [155,166]. A new carbapenemase (*bla*_OXA-497_) has been found in *A. baumannii* from dairy cattle [157]. A class 1 integron similar to an integron of a human isolate has been also identified in an equine *A. baumannii* isolate [167].

Currently, there are some reports underlying the emergence of *A. baumannii* strains resistant to both carbapenems and polymyxins (colistin and polymyxin B) in the clinical setting [168]. Resistance rates against polymyxins varied from 0.7% to 6.5% depending on the country [168]. Most polymyxin resistant strains from Europe have been recovered in Greece and Italy [168]. Interestingly, recent reports have also underlined the presence of colistin and polymyxin B resistant *A. baumannii* and other *Acinetobacter* spp. isolates in meat (chicken, turkey, beef and pork) [169,170]. Resistance to polymyxins in *A. baumannii* is mediated by mutations in the genes *pmrA* and/or *pmrB* [168]. Nevertheless, a new colistin resistance mechanism mediated by plasmids (the *mcr*-genes, see Section 5.3.4) has emerged in Enterobacteriaceae [168]. Although no clinical or environmental *A. baumannii* isolates carrying *mcr*-genes have yet been described, a recent study has proved that *A. baumannii* transformed with *mcr-1* carrying plasmids had increased colistin resistance [171]. This finding highlights the threat of a possible dissemination of these *mcr*-genes to multidrug resistant *A. baumannii* [171].

### 5.2. Pseudomonas aeruginosa

*P. aeruginosa* is an opportunistic pathogen often found in water and soil that is pathogenic to plants, humans, farm animals and companion animals. In humans, it is a cause of community and nosocomial infections, especially in patients immunocompromised and/or with cystic fibrosis. In animals, it caused pyoderma, otitis and urinary tract infections in companion animals, mastitis in dairy cows, endometritis in horses and hemorrhagic pneumoniae in fur-bearing animals [172,173,174,175,176]. Due to the presence of several drug efflux systems and porins, as well as its cell wall with low permeability, *P. aeruginosa* is intrinsically resistant to a wide range of antimicrobials including benzylpenicillins, aminobenzylpenicillins, carboxypenicillins, first and second generation cephalosporins, chloramphenicol and tetracycline [172,173]. Moreover, this bacterium is able to form biofilms and to acquire diverse resistance mechanisms.

The studies about AMR in animals are scarce, and mainly have focused in companion animals. A study has shown that *P. aeruginosa* isolates recovered from diverse veterinary samples during 1994–2003 have high resistance rates against β-lactams (70–100%) and sulphonamides (80–90%), while resistance against quinolones an aminoglycosides are more variable, with resistances ranging from 5 to 98% depending of the antibiotic [173]. Similar results were found in a study with canine isolates recovered between 2003 and 2006, although an increase in quinolone resistance was seen [174]. Resistance rates to the former antimicrobials are also similar in current studies among companion animals, although higher resistance rates against the aminoglycoside gentamicin [177] and the quinolone enrofloxacin [176] have been found. In *P. aeruginosa* from companion animals, the resistance to quinolones has been related to point mutations in *gyrA*, *gyrB*, *parC* and/or *parE* genes [172,176], and the resistance to aminoglycoside has been associated to diverse resistance genes (such as *aacA4* and *aadA6*) [172,176]. Resistance rates are generally lower in *P. aeruginosa* from livestock comparing to strains from companion animals [174,175].

Regarding its potential as zoonotic pathogen, a recent study by Haenni et al. [174] confirmed that the *P. aeruginosa* population in veterinary samples from France has a non-clonal epidemic structure. There was a poor association between an animal species and a specific clone, even though certain clones, possibly correlating with higher pathogenicity, seem to be more prevalent than others [174]. Clones associated to human outbreaks were detected, but not the most frequent epidemic clones associated to MDR in humans (also called “high risk clones”) [174].

The presence of ESBLs (via phenotypic methods) has been confirmed in *P. aeruginosa* isolates from animals [176,178]. Moreover, some reports have underlined the emergence of *P. aeruginosa* isolates with carbapenemases in animals (Table 6). The gene *bla*_IMP-4_ has been found in *P. aeruginosa* from dog [156], and the gene *bla*_VIM-2_ has been found in *P. aeruginosa* from cattle and fowl [151].

Similar to *A. baumannii*, recent reports have underlined the emergence of polymyxin-resistant *P. aeruginosa* [168]. Resistance rates are low (0.5% to 1.1%) in most countries, although, the situation is worrying in China where 22.2% of extensively drug-resistant bacteraemic *P. aeruginosa* isolates are resistant to polymyxin B [168]. Diverse mechanisms of polymyxin resistance have been described in *P. aeruginosa*, even though no clinical or environmental strains carrying *mcr*-genes have been reported [168]. Nevertheless, the recent study by Liu et al. [171] showed that *P. aeruginosa* isolates transformed with plasmids carrying *mcr-1* only had moderate changes in colistin susceptibility.

### 5.3. Enterobacteriaceae

As some Enterobacteriaceae (Ex. *Salmonella enterica*, *Yersinia enterocolitica*) are typically food-borne pathogens, this section will mainly focus on some transmissible AMRs-genes that have emerged in Enterobacteriaceae from animals (Table 7). The recent discover of the *cfr* gene in Enterobacteriaceae has been discussed at Section 4.3.2.

#### 5.3.1. Emergence of Streptothricin-Resistant *E. coli* in the 1980s

Similarly to Gram-positive bacteria, the use of antibiotics as grown promotors in the animal husbandry may potentiate the emergence of resistant Enterobacteriaceae isolates in food animals. A good example is the emergence of streptothricin-resistant *E. coli* in the 1980s. The aminoglycosidic growth promoter nourseothricin (streptothricin) was used in farm animals in Germany during the 1980s, while no equivalent antimicrobials were used in humans over this period [49]. Resistance emerged in *E. coli* from pigs the second year after the introduction of this antibiotic. It was mediated by a plasmid containing a transposon coding for a streptothricin acetyltransferase [181]. Subsequently, this resistance was found in *E. coli* isolated from pig farmers, in the community and in other Enterobacteriaceae from humans (*Salmonella* and *Shigella*) [49]. Nowadays, streptothricin-resistance is extensively extended and it is also characteristic of Gram-positive bacteria such as enterococci and staphylococci [182].

#### 5.3.2. ESBL/AmpC-Carrying Enterobacteriaceae in Animals

Bacteria carrying ESBLs are a worldwide clinical problem. ESBLs are mainly plasmid-encoded enzymes providing an extended resistance to β-lactam antibiotics, due to their ability to inactivate cephalosporins [49,183]. They can be produced by a variety of different bacteria including Enterobacteriaceae or no-fermenting bacteria (such as *P. aeruginosa*), *E. coli* and *K. pneumoniae* being the most frequently found ESBL-producing bacteria. ESBL-producing bacteria are known as nosocomial pathogens and since the late 1990s they have been increasingly found as a causal agent of infections in the community [183]. Until the 90s, the vast majority of ESBLs identified in human clinical isolates were SHV (sulfhydryl-variable) or TEM (named Temoneira for the first patient from whom the pathogen was isolated) types [184]. However, later, CTX-M (cefotaximase) β-lactamases have emerged and currently they are the most prevalent ESBLs in human Enterobacteriaceae [184].

The occurrence of ESBL-producing bacteria has been broadly recognized in veterinary medicine since the 2000s [183,184]. ESBL-producing bacteria have been found as disease agents and/or colonizers in livestock, companion animals, zoo animals and wild animals [54,183,184,185,186]. *E. coli* and *Klebsiella* spp. are cause of mastitis in dairy cattle, but most often livestock animals are asymptomatic carriers of ESBLs producers [54,187]. The first ESBL-producing *E. coli* was isolated from a dog with urinary tract infection and it carried SHV-12 [188]. Later on diverse CTX-M, TEM and SHV types have been observed in *E. coli*, *Salmonella* spp. and *K. pneumoniae* from livestock and companion animals [54,155,183,185,186]. The β-lactamases CTX-M-1, CTX-M-14, CTX-M-15, SHV-12 and TEM-52 are the most frequent types in Enterobacteriaceae from animals [54,155,183,185,186,187]. This distribution is similar in humans, where CTX-M types are the major β-lactamases in *E. coli* and *Klebsiella* spp. [54,183]. Host-range plasmids of different incompatibility groups (such as IncN, IncI, incF and IncK) have been related to these *bla*_CTX-M_ genes [154].

Identical phylogenic lineages (such as ST131) have been found in *E. coli* isolates from humans and animals [184]. The risk of zoonotic transfer from livestock to people with close contact to these animals is still largely unknown, but some studies have implicated a transfer of ESBL-producing *E. coli* or ESBLs genes from poultry or pigs to farm workers [183] Besides this direct zoonotic transfer, other routes as foods of animal origin may be a risk factor for human colonization or infection. In a recent study 70.6% of tested farms were ESBL-positive [183]. In one case the same isolate was detected in human and cattle samples, indicating a zoonotic transfer. In few other cases, pig and human isolates shared the same ESBLs genes, although the isolates belonged to different lineages suggesting horizontal gene transfer.

In contrast to the situation in Europe, ESBL genes have not been commonly reported in animal isolates in North America [154]. In North America, the plasmid-encoded AmpC B-lactamases genes are more frequently found [154], but these genes have also been described in Europe [54]. The broad-spectrum cephalosporinases (AmpC) were the first β-lactamases described in *E. coli* [47], and nowadays they have emerged worldwide [184]. As for ESBLs, *E. coli* carrying AmpC (*bla*_CMY-2_) has been identified in animals during the 2000s [184]. These β-lactamases have been detected in *E. coli* and *Salmonella* spp. from livestock and companion animals, being CMY-2 the most frequent one [184,185]. Other AmpC β-lactamases encoding by *bla*_ACC_ type genes have also been detected in *Salmonella* spp*.* from livestock [153,184].

#### 5.3.3. Carbapenemase-Producing Enterobacteriaceae in Animals

The carbapenemases are β-lactamases able of degrading carbapenems [153]. The epidemiologically most important carbapenemases are class B metallo-β-lactamases (MBLs) such as VIM (Verona integron-encoded MBL), IMP (imipenemase) and NDM (New Delhi MBL), class A, such as, KPC (*K. pneumoniae* carbapenemase), and class D including OXA (Carbapenem-hydrolysing oxacillinase) carbapenemases [153]. Resistance due to carbapenemases is mainly linked to the nosocomial setting, since carbapenems are not approved for use in veterinary medicine. However, they may be use for companion animals under certain conditions [154]. Although they are not used in livestock, carbapenem-resistant bacteria have been isolated from farm animals in recent years [154]. In animals, the first carbapenemase gene, *bla*_VIM-1_, was found in a porcine *E. coli* and it was linked to a multiresistance class 1 integron [152]. Later on, this and other carbapenemases have been found in livestock, wildlife and companion animals (Table 6), notably *bla*_VIM-1_ in *S. enterica* serovar Infantis and *E. coli* from livestock; *bla*_NDM-1_ in *S. enterica* sevorar Corvallis and *E. coli* from wild animals and/or livestock; *bla*_OXA-48_ in *E. coli* and *K. pneumoniae* from companion animals [153,155]. Carbapenemases have been detected in other Gram-negative bacteria including *A. baumannii* (see Section 5.1) and *P. aeruginosa* (see Section 5.2). However, the limited reports suggested that carbapenem-resistant bacteria are still at a very low prevalence in livestock [154].

#### 5.3.4. *mcr*-Genes Mediating Colistin Resistance

Colistin, also named polymyxin E, is currently used as a last-line drug against MDR Gram-negative bacteria [168,179]. However, resistance against colistin has even emerged in humans without contact to this antibiotic [189]. Moreover, since the 60s colistin has been used in pig production for therapeutic (in monotherapy), prophylactic and grown promotions purposes [190]. In addition, to its use in pigs, polymyxins (especially polymyxin B), are used in some countries for the treatment of coliform and *Pseudomonas* mastitis in cows [190]. Polymyxins are used in companion animals (dogs and cats) for topical indications such as otitis and ophthalmic diseases [190].

Colistin resistance is frequently due to chromosomal mutations and it has been detected in *P. aeruginosa*, *A. baumannii* and Enterobacteriaceae [168,179]. Most mechanisms conferring resistance against colistin are related to modifications of its primary target, the lipid A moiety of lipopolysaccharide (LPS) [168,179]. Colistin resistance mechanisms are different across bacterial genera, but most implied lipid A modifications with 4-amino-4-deoxy-l-arabinose (l-ara4N) and/or phosphoethanolamine (PEtN) [168,179].

The chromosomal-related colistin resistance mechanisms have no possibly of horizontal transfer, but plasmid-related genes (*mcr-1*, *mcr-2*) coding for PEtN transferases have recently emerged. The first *mcr*-gene, corresponded to *mcr-1*. It was initially described in an IncI2 plasmid from an *E. coli* from a pig, but in the same study it was soon after found in *E. coli* and *K. pneumoniae* from patients [191]. This gene was discovered in November 2015, but recent research has identified this gene in *E. coli* collections from the 80s [179]. The amino-acid sequence of MCR-1 showed that it was closely related (63%) to the PEtN transferase EptA found in *Paenibacillus sophorae* and *Enhydrobacter aerosaccus* [191]. Interestingly, the polymyxin is biosynthesized in *Paenibacillus* spp. [191]. Structural analysis of the MCR-1 complete protein showed that it was closely related to the PEtN transferases LptA of *Neisseria meningitidis* and EptC of *Campylobacter jejuni*, which are also known to be intrinsically resistant to polymyxin [179,191]. Further research showed that the prevalence of the *mcr-1* gene was 20% in animal strains and 1% in human strains in China, and nowadays it has been also detected in animal and human isolates recovered from other countries covering Europe, Africa and America [168,179,187].

The *mcr-1* gene has been identified in Enterobacteriaceae from humans, food, farm animals, wildlife, and environment samples [190]. Although it has been mainly detected (80%) in *E. coli*, it has also been described in *Klebsiella*, *Salmonella*, *Shigella* and *Enterobacter* [168,179]. In addition, it has recently been detected in *Cronobacter sakazakii* [192]. Interestingly, the *mcr-1* gene has been related to diverse plasmid incompatibility groups including IncFIA, IncFIB, IncFIC, IncFI, IncFII, IncHI2, IncI1, IncI2, IncN, IncP, IncQ1, IncX1, IncX4, IncY and pVT553 [168,179,190]. This gene has also been related to a class 1 integron within an IncFII plasmid [193]. Moreover, *mcr-1* has been identified in ESBL and carbapenamase producing Enterobacteriaceae from animals and humans (Table 8).

A variant (with only a SNP of difference) of *mcr-1*, *mcr-1.2* has been detected on a transferable IncX4 plasmid in a *K. pneumoniae* recovered from a patient [195]. This *mcr-1.2* strain also carried β-lactamases genes (*bla*_TEM-1_, *bla*_SHV-11_, and *bla*_KPC-3_) [195]. Very recently, a novel plasmid mediated colistin resistance gene, *mcr-2* has been discovered [198]. This *mcr-2* has been found in an IncX4 plasmid of *E. coli* from porcine and bovine origin in Belgium [198]. This gene corresponded to a new *mcr*-gene, since MCR-1 and MCR-2 shared 80.7% of similarity [198]. It has been seen that the *mcr-2* gene had higher prevalence than *mcr-1* in *E. coli* isolates from porcine origin [198].

Food animals seem the main source of human contamination by the *mcr*-genes [190]. However, the *mcr-1* gene has also been detected in *E. coli* carried or infecting humans without animal contact [190]. These findings underlined that this gene, which probably has emerged in the animal sector, is already widespread in the environment and it is transmissible via various routes to humans [190,199].

#### 5.3.5. Other AMR-Genes in Enterobacteriaceae from Animals

Increasing levels of quinolone resistance among Enterobacteriaceae and other bacteria (*Campylobacter* spp.) have been a particular case of concern since the 90s [155]. The extended use of quinolones to treat poultry infections has increased the quinolone resistance among *E. coli* strains in poultry industry [200]. Although quinolone resistance is generally not highly frequent in animal farming, high and/or moderate levels of quinolone resistance have been reported in poultry [200].

The mechanisms of quinolone resistance in Enterobacteriaceae from animals are similar to those described in isolates from humans [201]. Mutations at *gyrA* (DNA gyrase) and/or *parC* (topoisomerase IV) genes were responsible of quinolone resistance in *E. coli* and *Salmonella* spp. isolates from animals [155,201]. Moreover, reports about plasmid-mediated quinolone resistance (PMQR) genes [*qnr*, *acc(6)-Ib-cr*, *qepA*, *oqxAB*] in bacteria from animal origin have been published since 2000s [155,180].

The Qnr proteins are encoded by several variants of *qnr* genes (*qnrA*, *qnrB*, *qnrC*, *qnrD*, *qnrS*), being the *qnrS1* gene variant the most frequently reported and worldwide diffused in animal and human samples [200] (Table 9). Some *qnr* genes have been described in Enterobacteriaceae (mainly *Salmonella* spp. and *E. coli*) of animal origin [155,180,202]. Their wide distribution suggests an origin prior to the inclusion of quinolones in medicine [180]. It has been suggested that these genes have originated in bacteria from the natural environment [20]. In fact, *qnr* genes have been found in aquatic and waterborne organisms such as *Shewanella*, *Aeromonas* and *Citrobacter* species, and in the Vibrionaceae family [20,180].

As the *qnr* genes, PMQR efflux pump genes (*qepA* and *oqxAB*) have also a wide distribution, and they have been found in diverse Enterobacteriaceae from animals and humans [155,180] (Table 9). These genes are related to transmissible plasmids, although *oqxAB* is also commonly found in the chromosome of *K. pneumoniae* [180]. Other plasmid-mediated efflux pumps affecting quinolones have been described sporadically [180].

The *cr* variant of the aminoglycoside *aac(6′)-Ib* resistance gene was discover in 2006 [203]. The *aac(6′)-Ib* gene encodes a aminoglycoside acetyltransferase able to confer resistance against kanamycin, tobramycin and amikacin. The two mutations present at *aac(6′)-Ib-cr* confer low level ciprofloxacine resistance, with a slightly cost to the levels of aminoglycoside resistance [203]. Hence, it acts additively together with Qnr proteins to generate quinolone resistance [201]. This gene is widely distributed in Gram-negative bacteria (Table 9).

Regarding other aminoglycoside resistance genes, the gene *aac(3)-IV*, which confers cross-resistance between gentamicin and apramycin, need to be underlined [204]. It was originally isolated in 1981 from an *E. coli* recovered from farm animals in France. Although apramycin has only veterinary use, this gene has been detected in Enterobacteriaceae from human patients and wastewater from a residential area [204]. It has been suggested that apramycin consumption at farm level has increased the occurrence of *aac(3)-IV* positive *E. coli* in pigs [204].

Several other aminoglycoside resistance genes [such as *aac(3)-II*, *aac(3)-III*, *aadA1*, *aadA2*, *aadA5* and *ant(2″)-I*] have been described in *E. coli* from animal origin [200]. Other aminoglycoside resistance genes [*aac(3)-I*, *ant(2″)-Ia* and *aac(6)-Ib*] are more related to human *E. coli* isolates [200].

Enterobacteriaceae from animals carry other resistance genes. For example, a moderate incidence of chloramphenicol resistance is registered in *E. coli* from food animals in Europe, mainly mediated by genes such as *catA1*, *floR* and *cmlA1* [200]. Other chloramphenicol resistance genes (such as *catB*) are more frequent in human *E. coli* isolates [200].

*E. coli* isolates from animals carry also diverse sulfonamide (*sul1*, *sul2*, *sul3*), tetracycline [*tet*(A), *tet*(B)], and trimethoprim (*dfrA1*, *dfrA12*, *dfrA17*) resistance genes [200,204]. Interestingly, the gene *sul3* was first detected in an *E. coli* isolate from a pig, and later it was found in both healthy and diseased humans [204].

## 6. Conclusions

The continuous antibiotic selective pressure in human and animal health may contribute to the mobilization of acquired resistance genes. This is illustrated by several facts: (i) some studies have suggested that the *mecA* gene may have originated in animal related staphylococci; (ii) it has recently been observed that two mutiresistance genes (*cfr* and *optrA*) associated to MGEs such as plasmids along with other AMR-genes, have a wide dissemination in Gram-positive bacteria from animals and humans; (iii) typical nosocomial resistances linked to ESBLs and carbapenemases are emerging in Gram-negative bacteria from animals; and (iv) the *mcr*-genes, which may probably have emerged in the animal sector are currently spreading among human and animal *E. coli* isolates. These examples underline the fact that bacteria from animals represent an important pool of resistance genes for human pathogens.

Resistance to antibiotics is escalating, and at the same time the current pipeline of new antimicrobials is running dry, creating an ever increasing gap. Since a complete ban on the use of antimicrobials in farm animals would have serious repercussions for animal health, welfare and productivity, we need to use these agents more prudently in both human and animal medicine [3]. Rapid diagnosis tools are needed to determine therapy strategies more quickly and accurately, as well as the further examination of alternatives to antimicrobials for farm animals (such as phage therapy or vaccines) [3]. We are facing the possibility of a future without effective antibiotics for some infections and a scenario where infections that hitherto were considered harmless, are now a serious health problem and a major cause of morbidity, mortality, together with major financial and social repercussions.

## Figures and Tables

**Table 1:** Antimicrobials used in both human and veterinary medicine.

Group	Antimicrobial Agent(s)	Categorization in Human Medicine ^1^	Categorization in Veterinary Medicine ^2^
Aminoglycosides	Amikacin, dihydrostreptomycin, framycetin, gentamicin, kanamycin, neomycin, tobramycin, streptomycin	CIA	CIA
	Spectinomycin	IA	CIA
Ansamycins	Rifampicin, rifamixin	CIA	HIA ^4^
Cephalosporins (1st and 2nd generation)	Cefacetrile, cefalexin, cefalotin, cefapyrin, cefazolin, cefuroxime	HIA ^3^	HIA
Cephalosporins (3rd generation)	Cefoperazone, ceftriaxone	CIA	CIA
Macrolides	Erythromycin, oleandomycin, josamycin, spiramycin	CIA	CIA
Penicillins	Benzylpenicillin (penicillin G), amoxicillin, ampicillin, hetacillin, ticarcillin, phenoxymethylpenicillin (penicillin V), phenethicillin	CIA	CIA
	Cloxacillin, dicloxacillin, mecillinam, nafcillin, oxacillin	HIA ^3^	CIA
Penicillins + β-lactamase inhibitors	Amoxicillin-Clavulanic acid, Ampicillin-Sulbactam	CIA	CIA
Polymixins	Bacitracin	IA	HIA
	Colistin, polymyxin B	CIA	HIA
Quinolones (1st generation)	Flumequine, nalidixic acid, oxolinic acid	CIA	HIA
Quinolones (2nd generation)	Ciprofloxacin, norfloxacin, ofloxacin	CIA	CIA
Sulfonamides	Sulfadiazine, sulfadimethoxine, sulfadimidine, sulfafurazole (sulfisoxazole), sulfamerazine, sulfamethoxazole, sulfamethoxypyridazine, sulfanilamide, sulfapyridine	HIA ^3^	CIA
Tetracyclines	Chlortetracycline, doxycycline, oxytetracycline, tetracycline	HIA ^3^	CIA
Others	Fusidic acid	HIA ^3^	IA
	Fosfomycin	CIA	HIA ^4^
	Lincomycin	HIA	HIA
	Thiamphenicol	HIA ^3^	CIA
	Trimethoprim	HIA ^3^	CIA

^1^ Based on Anonymous [11]. ^2^ Based on Anonymous [13]. ^3^ In certain geographic settings this/these antimicrobial(s) can be considered CIA. ^4^ Only authorized in few countries and with a limited number of indications. CIA, critically important antimicrobial agent(s); HIA, highly important antimicrobial agent(s); IA, important antimicrobial agent(s).

**Table 2:** Examples of common AMR-genes found in *Clostridium*, Enterococci and Staphylococci.

Antimicrobial Agent(s) Group	Resistance Mechanism	Resistance Gene	Species Group
Chloramphenicol	Active efflux (MFS transporter)	*fexA*	Enterococci, Staphylococci
MLS_B_	Target site modification (rRNA methylation)	*erm*(A)	*Clostridium*, Enterococci, Staphylococci
		*erm*(B)	*Clostridium*, Enterococci, Staphylococci
Oxazolidinones	Active efflux (ABC transporter)	*optrA*	Enterococci, Staphylococci
PhLOPSA_A_	Target site modification (rRNA methylation)	*cfr*	Enterococci, Staphylococci
		*cfr*(B)	*Clostridium*, Enterococci
Tetracycline	Target site protection (ribosome protective protein)	*tet*(M)	*Clostridium*, Enterococci, Staphylococci
Trimethoprim	Target replacement (trimethoprim resistant dihydrofolate reductase)	*dfrK*	Enterococci, Staphylococci
Glycopeptides	Target replacement (modified peptidoglycan precursor)	*vanA*	Enterococci, Staphylococci

Based on Brenciani et al. [38], Deshpande et al. [39], He et al. [40], Fan et al. [41]; Liu et al. [42], Lopez et al. [43], Roberts et al. [44], Shen et al. [45], Spigaglia et al. [46], Van Hoek et al. [47] and Wendlandt et al. [48]. ABC, ATP-binding cassette; MFS, Major Facilitator Superfamily; MLS_B_, macrolide-lincosamide-streptogramin B; PhLOPSA_A_, phenicols, lincosamides, oxazolidinones, pleuromutilins and streptogramin A.

**Table 3:** Examples of AMR-genes identified in Staphylococci from animal and/or human origin.

Antimicrobial Agent(s) Group	Resistance Mechanism	Resistance Gene(s)	Staphylococci Origin
Β-lactams	Enzymatic inactivation (hydrolization)	*blaZ*	A, H
	Target site replacement (alternative PBP)	*mecA*, *mecC* (*mecA*_LGA251_)	A, H
Aminoglycosides	Enzymatic inactivation (acetylation and phosphorylation)	*aacA-aphD*	A, H
	Enzymatic inactivation (adenylation)	*aadD*, *aadE*, *str*	A, H
	Enzymatic inactivation (phosphorylation)	*aphA3*	A, H
Aminocylitols	Enzymatic inactivation (adenylation)	*spc*, *spd*, *spw*	A, H
	Enzymatic inactivation (acetylation)	*apmA*	A
Bleomycin	Bleomycin binding protein	*ble*	A, H
Fosfomycin	Enzymatic inactivation (metallothiol-transferase)	*fosD* (*fosB)*	A, H
Fusidic acid	Target site protection (ribosome protective protein)	*fusB*, *fusC*	A, H
Macrolides	Active efflux (MFS transporter)	*mef(A)*	H
	Enzymatic inactivation (phosphorylation)	*mph(C)*	A, H
Macrolides, streptogramin B	Active efflux (ABC transporter)	*msr*(A)	A, H
MLS_B_	Target site modification (rRNA methylation)	*erm*(A), *erm*(B), *erm*(C), *erm*(F), *erm*(T), *erm*(43)	A, H
		*erm*(33), *erm*(44), *erm*(45)	A
		*erm*(G), *erm*(Q), *erm*(Y), *erm*(44)v	H
Mupirocin	Target replacement (mupirocin-insensitive isoleucyl-tRNA synthase)	*mupA* (*ileS2*)	A, H
		*mupB*	H
Lincosamides	Enzymatic inactivation (nucleotidylation)	*lnu*(A), *lnu*(B)	A, H
	Active efflux (ABC transporter)	*lsa*(B)	A
Lincosamides, streptogramin A	Active efflux (ABC transporter)	*sal(A)*	A
LPS_A_	Active efflux (ABC transporter)	*vga*(A), *vga*(A)v, *lsa*(E)	A, H
		*vga(B)*	H
		*vga*(C), *vga*(E), *vga*(E)_variant_	A
Phenicols	Enzymatic inactivation (acetylation)	*cat*_pC221_, *cat*_pC223_, *cat*_pC194_	A, H
	Active efflux (MFS transporter)	*fexA*	A, H
PhLOPSA_A_	Target site modification (rRNA methylation)	*cfr*	A, H
Streptogramin A	Enzymatic inactivation (acetylation)	*vat*(A)	H
		*vat*(B)	A, H
		*vat*(C)	H
Streptogramin B	Enzymatic inactivation (hydrolization)	*vgb*(A)	H
		*vgb*(B)	A, H
Streptothricins	Enzymatic inactivation (acetylation)	*sat4*	A, H
Tetracyclines	Active efflux (MFS transporter)	*tet*(K), *tet*(L)	A, H
	Target site protection (ribosome protective protein)	*tet*(M)	A, H
		*tet*(O)	A
Oxazolidinones-phenicols	Active efflux (ABC transporter)	*optrA*	A
Trimethoprim	Target replacement (trimethoprim resistant dihydrofolate reductase)	*dfrA* (*dfrS1*), *dfrD*, *dfrG*, *dfrK*	A, H
Vancomycin	Target replacement (modified peptidoglycan precursor)	*vanA*	H

Based on Argudín et al. [82,84,92,93], Fan et al. [41], Li et al. [68], Schwarz et al. [80], Seah et al. [94], Strauss et al. [95], Wendlandt et al. [48,81] and Wipf et al. [96,97]. A, animal origin; ABC, ATP-binding cassette; H, human origin; LPS_A_, lincosamides-pleuromutilins-streptogramin A; MFS, Major Facilitator Superfamily; MLS_B_, macrolide-lincosamide-streptogramin B; PBP, penicillin binding protein; PhLOPSA_A_, phenicols, lincosamides, oxazolidinones, pleuromutilins and streptogramin A.

**Table 4:** Examples of the genetic environments of *cfr* genes.

Genetic Environment	Strain or Plasmid Name	Accession Number	Species	Additional Resistance Genes
Chromosomal region	Strain CM05	JN849634	*S. aureus*	*erm*(B)
	Strain FSEC-02	KR779900	*E. coli*	-
	Strain Ox3196 (*Tn*6218)	HG002389	*C. difficile*	-
	Strain PV-01	JF969273	*P. vulgaris*	-
Plasmid	P3-38	JQ911740	*E. thailandicus*	-
	p004-737X	EU598691	*S. aureus*	-
	p7LC	JX910899	*S. epidermidis*	*aacA–aphD*
	pBD-01	GU591497	*S. cohnii*	*erm*(B)
	pBS-01	GU591497	*Bacillus* spp.	*erm*(B)
	pBS-02	HQ128580	*Bacillus* spp.	-
	pBS-03	JQ394981	*Bacillus* spp.	*aadY*
	pEC-01	JN982327	*E. coli*	-
	pEF-01 ^1^	NC_014508	*E. faecalis*	*fexB*
	pERGB	JN970903	*S. aureus*	*aadD*, *tet*(L), *dfrK*
	pFSEC-01	KR779901	*E. coli*	-
	pGXEC3	KM580532	*E. coli*	*bla*_CTX-M-14b_
	pGXEC6	KM580533	*E. coli*	-
	pHNEP28	KT845955	*E. coli*	-
	pHOU-*cfr*	JQ660368	*E. faecalis*	-
	pMHZ	JX232067	*S. capitis*	-
	pMSA16	JQ246438	*S. aureus*	*erm*(A)
	pSCEC2	KF152885	*E. coli*	*floR*, *tet*(A)-*tetR*, *strA/str*, *sul*
	pSS-01	JQ041372	*S. cohnii*	*aacA–aphD*, *fexA*
	pSS-02	JF834910	*S. saprophyticus*	*fexA*
	pSS-03	JQ219851	*S. cohnii*, *M. caseolyticus*	*erm*(C)
	pSCFS1	NC_005076	*S. sciuri*	*erm*(33), *lsa*(B), *spc*
	pSCFS3	AM086211	*S. aureus*	*fexA*
	pSCFS6	AM408573	*S. warneri*	*fexA*, *lsa*(B)
	pSCFS7	FR675942	*S. aureus*	*fexA*
	pSD11	KM212169	*E. coli*	-
	pSP01	KR230047	*S. epidermidis*	*blaZ*, *lsa(B)*, *msr*(A), *aad*
	pStrcfr	KC844846	*S. suis*	∆*lnu*(E)
	pJP1	JQ320084	*J. pinnipedialis*	*aadD*, *ble*
	pJP1-like	KF129408	*S. lentus*	*aacA–aphD*, *aadD*, *ble*, *fexA*
	pJP2	KC989517	*S. rostri*	*aacA–aphD*, *aadD*, *ble*, *fexA*, *fosD*
	pW3	JQ911739	*E. thailandicus*	*erm*(B)
	pW9-2	JQ911741	*E. faecalis*	-
	pWo28-3	KT601170	*S. sciuri*	*aacA-aphD*, *aadD*, *ble*, *fexA*, *optrA*
Possible plasmids	Strains UW10882 and UW12712 (*Tn*6218-like)	SRP078305	*E. faecium*	-

Based on Bender et al. [128], Brenciani et al. [129], Gopegui et al. [121], Li et al. [68], Liu et al. [130], Shen et al. [45], Sun et al. [131], Wang et al. [126], Wendlandt et al. [81] and Zhang et al. [132,133,134]. ^1^ *cfr*-carriying plasmids related to pEF-01 have been found in *E. casseliflavus* and *E. gallinarum* [42].

**Table 5:** Examples of resistance genes co-located on plasmids from Staphylococci.

**AAG**	**Gene(s)**	**Co-Location with:**																											
		*apmA*	*spc*	*aacA-aphD*	*aadD*	*blaZ*	*ble*	*fosD*	*cadXD*	*copA*	*mco*	*erm*(A)	*erm*(B)	*erm*(C)	*erm*(T)	*erm*(33)	*lnu*(B)	*lsa*(B)	*lsa*(E)	*vga*(A)	*vga*(C)	*fexA*	*cfr*	*tet*(K)	*tet*(L)	*tet*(M)	*optrA*	*dfrK*
AC	*apmA*	/			X				X	X	X		X												X			X
	*spc*		/													X		X					X					
AG	*aacA-aphD*			/	X	X	X	X	X				X				X		X			X	X	X	X		X	
	*aadD*	X		X	/		X	X	X	X	X		X		X					X	X	X	X		X	X	X	X
BL	*blaZ*			X		/		X	X				X					X					X					
BM	*ble*			X	X		/	X														X	X				X	
FM	*fosD*			X	X	X	X	/	X														X					
HM	*cadXD*	X		X	X	X		X	/	X	X		X	X	X		X								X			X
	*copA*	X			X				X	/	X		X	X	X										X			X
	*mco*	X			X				X	X	/		X	X	X										X			X
MLS_B_	*erm*(A)											/											X					
	*erm*(B)	X		X	X	X			X	X	X		/				X		X			X	X		X			X
	*erm*(C)								X	X	X			/	X								X	X				
	*erm*(T)				X				X	X	X			X	/										X			X
	*erm*(33)		X													/		X					X					
LN	*lnu(B)*			X					X				X				/		X						X			
LPS_A_	*lsa*(B)		X			X							X			X		/				X	X					
	*lsa*(E)			X													X		/						X			
	*vga*(A)				X															/					X	X		X
	*vga*(C)				X																/				X			X
Ph	*fexA*			X	X		X						X					X				/	X				X	
PhLOPSA_A_	*cfr*		X	X	X	X	X	X				X	X	X		X		X				X	/		X		X	X
TC	*tet*(K)			X										X										/				
	*tet*(L)	X		X	X				X	X	X		X		X		X		X	X	X		X		/	X		X
	*tet*(M)				X															X					X	/		X
OP	*optrA*			X	X		X															X	X				/	
TM	*dfrK*	X			X				X	X	X		X		X					X	X		X		X			/

A black square indicates that the corresponding resistance gene(s) can be found co-located together in plasmids. This table is based on the plasmids described by Fan et al. [41], Gómez-Sanz et al. [148], Gopegui et al. [121], Kadlec et al. [79], Li et al. [68], Shen et al. [45], Schwarz et al. [78,80] and Wendlandt et al. [81]. AAG, antimicrobial agent(s) group; AC, aminocylitols; AG, aminoglycosides; BL, Β-lactams; BM, bleomycin; FM, fosfomycin; HM, heavy metals; LN, lincosamides; LPS_A_, lincosamides-pleuromutilins-streptogramin A; MLS_B_, macrolide-lincosamide-streptogramin B; OP, oxazolidinones-phenicols; Ph, phenicol; PhLOPSA_A_, phenicols, lincosamides, oxazolidinones, pleuromutilins and streptogramin A; TC, tetracycline; TM, trimethoprim.

**Table 6:** Examples of carbapenemase genes found in Gram-negative bacteria from animals.

Gene	Species	Origin
*bla*_IMP-4_	*P. aeruginosa*	Dog
*bla*_NDM-1_	*A. baumannii*	Pig
	*E. coli*, *S. enterica*	Various livestock and wildlife animals
*bla*_NDM-5_	*E. coli*	Fowl
*bla*_NDM-9_	*E. coli*	Chicken
*bla*_OXA-23_	*A. baumannii*	Various livestock and companion animals
	*A. lwoffi*	Poultry
*bla*_OXA-48_	*E. coli*, *K. pneumoniae*	Companion animals
*bla*_OXA-58_	*A. baumannii*	Fowl
*bla*_OXA-497_	*A. baumannii*	Dairy cattle
*bla*_VIM-1_	*E. coli*, *S. enterica*	Various livestock, companion and wildlife animals
*bla*_VIM-2_	*P. aeruginosa*	Cattle, fowl

Based on Al Bayssari et al. [151], Fisher et al. [152], Guerra et al. [153], Michael et al. [154], Schwarz et al. [155], Wang et al. [156], Webb et al. [157] and Zhang et al. [158].

**Table 7:** Examples of important plasmid-associated resistance mechanisms in Enterobacteriaceae from animals.

Antimicrobial Group	Resistance Mechanism	Example Gene(s)
Aminoglycosides	Enzymatic inactivation (acetylation)	*acc(3)-IV*
Aminoglycosides/Quinolones	Enzymatic inactivation (acetylation)	*aac(6′)-Ib-cr*
β-lactams	Enzymatic inactivation (hydrolization)	*bla_CTX-M_*
Quinolones	Target replacement (pentapeptide repeat protein)	*qnr*
	Active efflux (MFS transporter)	*qepA*
	Active efflux (RND transporter)	*oqxAB*
PhLOPSA_A_	Target site modification (rRNA methylation)	*cfr*
Polymyxins	Target site modification (PEtN transferase)	*mcr*

Based on Baron et al. [179], Rodríguez-Martínez et al. [180] and Shen et al. [45]. MFS, Major Facilitator Superfamily; PEtN, phosphoethanolamine; PhLOPSA_A_, phenicols, lincosamides, oxazolidinones, pleuromutilins and streptogramin A; RND, resistance-nodulation-cell division family.

**Table 8:** Associated β-lactam resitances in colistin-resistant *mcr-1* carrying isolates.

Species	Origin	Associated β-Lactam Resitances		
		ESBLs	AmpC	Carbapenemases
*C. sakazakii*	A	-	-	NDM-9
*E. coli*	A	CTX-M-1, CTX-M-2, CTX-M-8, CTX-M-15, CTX-M-27, CTX-M-55, TEM-1	CMY-2, LAT-1	NDM-1, NDM-5, NDM-9
	F	CTX-M-1, CTX-M-14, CTX-M-15, CTX-M-55, CTX-M-65, SHV-12, TEM-1, TEM-52	CMY-2	NDM-9, OXA-1
	H	CTX-M-1, CTX-M-2, CTX-M-8, CTX-M-14, CTX-M-15, CTX-M-27, CTX-M-55, CTX-M-65, SHV-12, TEM-1, TEM-52	ACT-15, CMY-2, DHA-1	KPC-2, NDM-1, NDM-5, OXA-1, OXA-48, VIM-1
	E	SHV-12, TEM-1	-	-
	W	CTX-M-2, CTX-M-14	-	-
*Enterobacter* spp.	H	CTX-M-15, TEM-1	-	KPC-2, OXA-1
*K. pneumoniae*	H	CTX-M-1, SHV-11, TEM-1	-	KPC-3, NDM-5
*S. enterica*	A	TEM-1	-	-
	F	CTX-M-1, TEM-1	-	-
	H	TEM-1	CMY-2	-

Based on Delgado-Blas et al. [194], Di Pilato et al. [195], Jeannot et al. [168], Kong et al. [196], Liu et al. [192] and Mediavilla et al. [197]. A, animal origin; E, environmental origin; F, food origin; H, human origin; W, wildlife origin.

**Table 9:** Examples of distribution of certain PMQR-genes.

Gene	Species	Origin
*acc(6)-Ib-cr*	*Aeromonas* spp.	E, W
	*C. freundii*, *C. koseri*, *Enterobacter* spp., *P. aeruginosa*, *P. mirabilis*, *Stenothrophomonas maltophilia*, *Shigella* spp.	H
	*E. coli*	C, E, F, H, L, W, Z
	*Haemophilus parasuis*	L
	*K. pneumoniae*	C, H, Z
	*Laribacter hongkongensis*	E, W
	*Salmonella* spp.	E, F, H, L, W
*oqxAB*	*E. coli*	E, F, H, L, W, Z
	*K. pneumoniae*	H
	*Salmonella* spp.	F, H, L
*qepA*	*E. coli*	C, E, H, L
	*K. pneumoniae*	H
	*Salmonella* spp.	F, H
	*Shigella* spp.	H, E
*qnrA1*	*C. freundii*, *E. cloacae*, *K. pneumoniae*, *P. aeruginosa*, *P. mirabilis*	H
	*E. coli*	C, L
	*H. parasuis*	L
	*Salmonella* spp.	H, L
*qnrA3*	*Shewanella algae*	E
*qnrA6*	*E. coli*, *K. pneumoniae*, *Morganella morganii*, *P. mirabilis*	H
*qnrB1*	*C. freundii*, *K. pneumoniae*	H
	*E. coli*	H, W
*qnrB2*	*C. freundii*, *K. pneumoniae*	H
	*E. coli*	C, H, L
	*Salmonella* spp.	E, H, L
*qnrB4*	*C. freundii*	H
	*E. coli*	L
	*Salmonella* spp.	H, L
*qnrB5*	*Salmonella* spp.	F
*qnrB6*	*E. coli*	E, L
	*H. parasuis*	L
	*K. pneumoniae*	H
	*Salmonella* spp.	W
*qnrB7*	*Salmonella* spp.	H
*qnrB8*-variant	*C. freundii*	H
*qnrB9*	*C. freundii*	E
*qnrB10*	*C. freundii*, *K. pneumoniae*	H
	*E. coli*	L
*qnrB12*	*Salmonella* spp.	H, L
*qnrB17*	*Aeromonas* spp.	E, W
	*E. coli*	L
*qnrB19*	*E. coli*	C, E, H, L
	*K. pneumoniae*	H
	*Salmonella* spp.	F, H, L, W
*qnrB24*	*C. freundii*	H
*qnrD*	*E. coli*	E, L
	*P. mirabilis*	C, H, E
	*Salmonella* spp.	F, H, L
*qnrS1*	*E. coli*	C, E, F, H, L, W, Z
	*Enterobacter* spp.	E
	*K. pneumoniae*, *Shigella* spp.	H
	*Salmonella* spp.	E, F, H, L, W
*qnrS2*	*Aeromonas* spp.	E, W
	*E. coli*	F
	*Pseudomonas* spp., *Pseudoalteromonas* spp.	E
*qnrS5*	*Aeromonas* spp.	E, W

Based on Rodriguez–Martinez et al. [180], Schwarz et al [155] and Veldman et al. [202]. C, companion animals origin; E, environmental origin; F, food origin; H, human origin; L, livestock origin; W, wildlife origin; Z, zoo animals.

## Abbreviations

l-ara4N	4-amino-4-deoxy-l-arabinose
ABC	ATP-binding cassette
AMR	antimicrobial resistance
CC	clonal complex
CoNS	coagulase negative staphylococci
CTX-M	cefotaximase
ECDC	European Centre for Disease Prevention and Control
EFSA	European Food Safety Authority
*erm*	erythromycin ribosomal methylase
ESBLs	extended spectrum beta-lactamases
ESC(K)APE	*E. faecium, S. aureus, Clostridium difficile, A. baumannii, P. aeruginosa,* and Enterobacteriaceae
ESKAPE	*Enterococcus faecium, S. aureus, Klebsiella pneumoniae, Acinetobacter baumannii, Pseudomonas aeruginosa,* and *Enterobacter* spp.
HIV	human immunodeficiency virus
IMP	imipenemase
IS	insertion sequence
KPC	*K. pneumoniae* carbapenemase
QRDR	quinolone-resistance determining region
LA-MRSA	livestock-associated methicillin resistant *Staphylococcus aureus*
LPS	lipopolysaccharide
MBL	metallo-β-lactamase
MFS	Major Facilitator Superfamily
MGE	mobile genetic element
MLS_B_	macrolide-lincosamide-streptogramin B
MLST	multilocus sequence typing
MRCoNS	methicillin resistant coagulase negative staphylococci
MDR	multidrug resistance
MRSA	methicillin resistant *Staphylococcus aureus*
MRSE	methicillin-resistant *Staphylococcus epidermidis*
MSSA	methicillin susceptible *S. aureus*
NDM	New Delhi metallo-β-lactamases
OIE	World Organization for Animal Health
OXA	carbapenem-hydrolysing oxacillinase
PBP	penicillin binding protein
PCR	polymerase chain reaction
PEtN	phosphoethanolamine
PFGE	pulsed-field gel electrophoresis
PMQR	plasmid-mediated quinolone resistance
PVL	Panton-Valentine leucocidin
REA	restriction enzyme analysis
RND	resistance-nodulation-cell division family
RT	ribotype
SCC*mec*	staphylococcal cassette chromosome *mec*
SHV	sulfhydryl-variable β-Lactamase
SNP	single nucleotide polymorphism
ST	sequence type
TEM	Temoneira β-Lactamase
VIM	Verona integron-encoded metallo-β-lactamase
VRE	vancomycin-resistant enterococci
WHO	World Health Organization
